# Anomalous Origin of a Right Coronary Artery from Pulmonary Artery

**DOI:** 10.1155/2018/2583918

**Published:** 2018-12-19

**Authors:** Muhammad Shabbir Rawala, S. Tahira Shah Naqvi, Kinaan Farhan, Muhammad Yasin, Syed Bilal Rizvi

**Affiliations:** ^1^Department of Internal Medicine, WVU-Charleston Division, Charleston, WV, USA; ^2^Department of Medicine, Jinnah Medical & Dental College, Karachi, Pakistan; ^3^Department of Cardiology, Rapides Regional Medical Center, Alexandria, LA, USA

## Abstract

Congenital defects of the coronary arteries are noted in 0.2–1.4% of the general population. The first case of an anomalous origin of right coronary artery from pulmonary artery (ARCAPA) was described by Brooks in 1885. ARCAPA has an overall incidence of 0.002% in the general population. Most of the cases are asymptomatic; however, it can lead to serious complications such as heart failure, ischemia, and sudden death. A 57-year-old man presented to the cardiologist's office with complaints of shortness of breath and fatigue. The patient also had a previous history of coronary stents and heart failure. Initially, he was evaluated with a stress test which was reported as abnormal. The patient then underwent an invasive coronary angiography that revealed anomalous origin of the right coronary artery (RCA) and multivessel disease. Cardiothoracic surgery evaluated the patient and coronary artery bypass graft was performed. During the surgery, the anomalous origin of RCA from the pulmonary artery was identified and was successfully corrected by reimplanting the RCA into the ascending aorta. The anomalous origin of RCA is a rare yet life-threatening condition. The RCA due to its location of origin from the pulmonary artery tends to be a low-pressure vessel with a very thin and fragile wall. It also serves as a retrograde venous conduit from the left coronary circulation into the pulmonary artery. This connection results in a left-to-right shunt that explains the increase in oxygen saturation in the pulmonary artery and the high cardiac output which is normally seen in these cases. The clinical presentation can vary from coronary ischemia to heart failure or sudden death. Therefore, surgical correction is recommended even in asymptomatic patients. We present a case of an anomalous origin of RCA from the pulmonary artery which, unlike the origin of left coronary from pulmonary artery, is very rare. Patients with this condition should have early correction even if they are asymptomatic in order to prevent long-term complications.

## 1. Introduction

Anomalous origin of the right coronary artery from the pulmonary artery (ARCAPA) is a rare congenital defect [1]. Brooks described the first case of ARCAPA in 1885 [2]. ARCAPA is potentially life threatening due to its consequential conditions such as coronary ischemia, heart failure, and sudden cardiac death (in infancy/adolescence/adulthood due to fatal arrhythmia) [3]. This defect is also known to pose as a challenge during surgical procedures, usually because it is incidentally diagnosed in the operation room [4]. Most patients with ARCAPA are asymptomatic and sometimes the anatomical defect may be subtle and thus difficult to visualize. For these reasons, patients may not be diagnosed until the disease is highly advanced, or oftentimes, it may only be detected during a postmortem examination [5, 6]. Many different imaging modalities are available to diagnose ARCAPA. These include echocardiography, magnetic resonance angiogram (MRA), and multislice-gated coronary CT angiogram, with the latter two being more reliable [3]. The detection of ARCAPA is usually incidental while patient is undergoing evaluation for other problems, for example, a coronary angiography being done for chest pain [7]. Because of its high-risk profile, surgical treatment of ARCAPA is strongly recommended, even for asymptomatic patients [8].

## 2. Case Presentation

**Figure 1:**
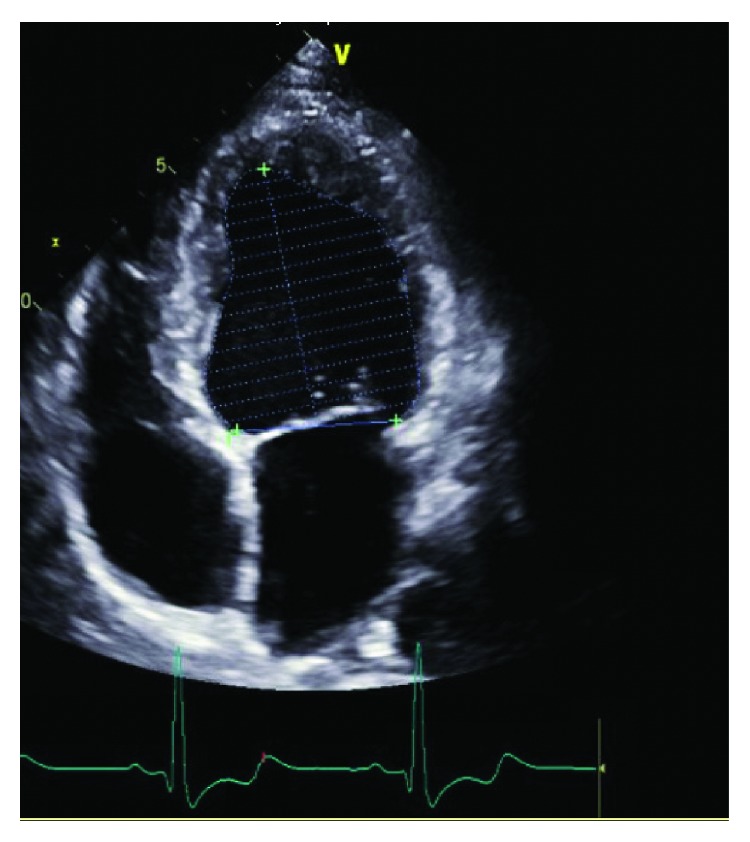
Echocardiogram showing moderate dilation of the left ventricle and mildly reduced ejection fraction.

**Figure 2:**
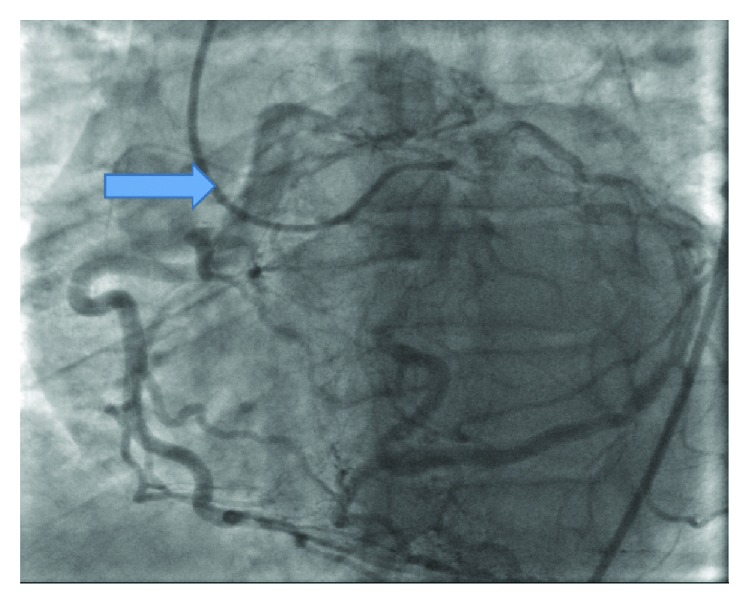
Catheterization showing the posterior descending artery and posterior left ventricular artery branches of the right coronary artery from the pulmonary artery (arrow).

**Figure 3:**
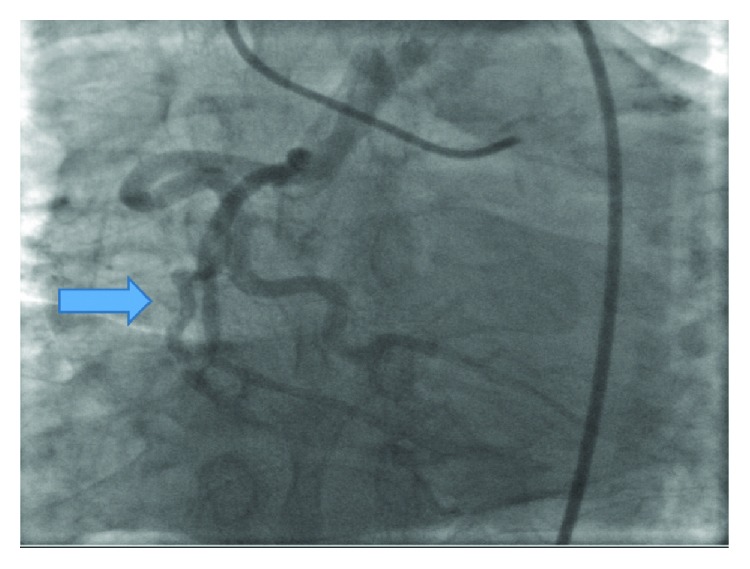
Catheterization showing the posterior descending artery and posterior left ventricular branches of the right coronary artery.

A 57-year-old man presented to the cardiologist's office as a part of the preoperative evaluation before undergoing an ophthalmological surgery. He complained of shortness of breath and fatigue. The patient had a prior history of coronary stents and heart failure. Physical examination was unremarkable. Echocardiogram reported moderate dilation of the left ventricle and hypokinesis of the apical lateral wall and apical septal wall. Ejection fraction was reported to be 35-40% (Figure 1). The patient was evaluated by a nuclear stress test which came out to be abnormal. The patient then underwent an invasive coronary angiography that showed a 70% eccentric obstruction of the proximal left anterior descending (LAD) artery and a 70% obstruction of the left circumflex artery as well with diffuse calcification. The right coronary artery (RCA) was believed to be anomalous (Figures 2 and 3). Cardiothoracic surgery evaluated the patient and coronary artery bypass graft was performed that identified intraoperatively an anomalous origin of RCA from the pulmonary artery which was then successfully reimplanted into the ascending aorta.

## 3. Discussion

Congenital coronary artery anomalies are known to be rare in the general population. The incidence of isolated ARCAPA is only 0.3%–0.9%, with the rate being up to 36% in patients with other associated congenital heart anomalies [9]. The congenital coronary artery defects described in the literature mostly include the left coronary artery originating from the pulmonary artery. There are very few case reports describing the ARCAPA [4]. ARCAPA represents 0.12% of all coronary anomalies and its overall incidence is estimated to be 0.002% in the general population [1]. Many cases are asymptomatic and undiagnosed; hence, the true prevalence of ARCAPA is likely underestimated [9].

The most common cardiac defects reported to be associated with ARCAPA were tetralogy of Fallot and aortopulmonary window. Other relatively less common ones include aortic stenosis, septal defects, and aortic coarctation [1].

ARCAPA is known to be associated with a risk of sudden cardiac death. A great number of cases were diagnosed postmortem, including individuals from various age groups in whom the pathomorphological features indicated cardiac muscle necrosis [6, 8]. While most cases of ARCAPA, as previously mentioned, are asymptomatic, some patients may be symptomatic with the common presenting signs/symptoms being murmur, angina, dyspnea on exertion, and congestive heart failure [10].

ARCAPA is generally a well-tolerated condition in the neonatal period. The high pulmonary vascular resistance in neonates allows forward perfusion of the anomalous RCA from the pulmonary artery. Eventually, as the pulmonary vascular resistance starts to fall, the well-oxygenated blood coming into the anomalous coronary (through the collateral vessels from the left coronary) is forwarded to the pulmonary trunk. This results in a “steal phenomenon” causing chronic myocardial ischemia and symptoms related to chronic left-to-right shunt. This may also result in ventricular dilatation and dysfunction with the severity depending on the shunt size, the number of collaterals, the territory at risk, and the overall myocardial oxygen demands [11, 12].

ARCAPA patients may have a normal EKG or they may have an EKG showing deep Q-waves in the inferior leads or anomalies indicating left ventricular hypertrophy. The top diagnostic modalities for ARCAPA include cardiac computed tomography, coronary angiogram, and cardiovascular magnetic resonance, all of which provide excellent visualization of the coronary artery and the anomalies with detailed information regarding the origin, anatomic relationship, and course of the anomalous coronary artery [8].

Patients who are not surgically treated for this anomaly have been seen to typically have a shorter lifespan which is why immediate surgical treatment (as soon as the condition is diagnosed) is strongly recommended [5]. Surgical correction of ARCAPA is associated with low postoperative mortality, ranging from 2% to 3% [9]. The aim of surgical correction is (I) eliminate the left-to-right shunt and (II) to establish dual coronary circulation. This prevents the potential risk of myocardial ischemia resulting from coronary steal phenomenon. The technique used for surgical repair is influenced by the location of the ostium of the right coronary artery in the pulmonary artery [9, 13].

There are various surgical techniques to treat ARCAPA, the most common one being reimplanting the RCA into the right aortic sinus. Other approaches include complete ligation of RCA proximally with saphenous vein graft between the aorta and distal RCA. Another technique is the Takeuchi procedure which is mostly performed in children. It consists of creating an aortopulmonary window and the flow between the RCA ostium and the aorta is established using an intrapulmonary tunnel [8, 14, 15].

## 4. Conclusion

This case highlights a rare condition of the right coronary artery originating anomalously from the pulmonary artery that can lead to sudden cardiac death. Surgery to correct the defect must be done even in asymptomatic patients to prevent risk of sudden death and other complications arising from ARCAPA.

Our patient did have a history of prior coronary stents placed; however, the procedure was not done at our facility, nor did the patient know of the abnormality; therefore, the correction of ARCAPA was not pursued earlier.
